# Highly accelerated, Dixon-based non-contrast MR angiography versus high-pitch CT angiography

**DOI:** 10.1007/s11547-023-01752-0

**Published:** 2023-11-29

**Authors:** Martin Georg Zeilinger, Daniel Giese, Michaela Schmidt, Matthias Stefan May, Rolf Janka, Rafael Heiss, Fabian Ammon, Stephan Achenbach, Michael Uder, Christoph Treutlein

**Affiliations:** 1 Institute of Radiology, University Hospital of Erlangen, Friedrich-Alexander-Universität Erlangen-Nürnberg, Erlangen, Germany; 2 Magnetic Resonance, Siemens Healthcare, Erlangen, Germany; 3 Institute of Cardiology, University Hospital of Erlangen, Friedrich-Alexander-Universität Erlangen-Nürnberg, Erlangen, Germany

**Keywords:** Magnetic resonance imaging, Computed tomography, Angiography, Follow-up studies

## Abstract

**Objectives:**

To compare a novel, non-contrast, flow-independent, 3D isotropic magnetic resonance angiography (MRA) sequence that combines respiration compensation, electrocardiogram (ECG)-triggering, undersampling, and Dixon water-fat separation with an ECG-triggered aortic high-pitch computed tomography angiography (CTA) of the aorta.

**Materials and methods:**

Twenty-five patients with recent CTA were scheduled for non-contrast MRA on a 3 T MRI. Aortic diameters and cross-sectional areas were measured on MRA and CTA using semiautomatic measurement tools at 11 aortic levels. Image quality was assessed independently by two radiologists on predefined aortic levels, including myocardium, proximal aortic branches, pulmonary veins and arteries, and the inferior (IVC) and superior vena cava (SVC). Image quality was assessed on a 5-point Likert scale.

**Results:**

All datasets showed diagnostic image quality. Visual grading was similar for MRA and CTA regarding overall image quality (0.71), systemic arterial image quality (*p* = 0.07–0.91) and pulmonary artery image quality (*p* = 0.05). Both readers favored MRA for SVC and IVC, while CTA was preferred for pulmonary veins (all *p* < 0.05). No significant difference was observed in aortic diameters or cross-sectional areas between native MRA and contrast-enhanced CTA (*p* = 0.08–0.94).

**Conclusion:**

The proposed non-contrast MRA enables robust imaging of the aorta, its proximal branches and the pulmonary arteries and great veins with image quality and aortic diameters and cross-sectional areas comparable to that of CTA. Moreover, this technique represents a suitable free-breathing alternative, without the use of contrast agents or ionizing radiation. Therefore, it is especially suitable for patients requiring repetitive imaging.

## Introduction

Diseases of the aorta are among the leading causes of morbidity and mortality in Western countries [1]. They include aortic aneurysms, acute aortic syndromes such as aortic dissection, atherosclerotic lesions, inflammatory diseases, and genetic and congenital abnormalities, such as Marfan or Loeys-Dietz syndrome and malformations of the aortic arch [2]. While some of these conditions require immediate surgical or interventional therapy, many cases also require repeated follow-up imaging [2]. Early detection of vascular changes is essential for timely interventions, including increased aortic aneurysm diameter, dissection, penetrating aortic ulcers prone to rupture, or aortic coarctation grading [3, 4].

Repetitive imaging is mandatory during follow-up and after endovascular aortic repair or open surgery, and several imaging methods are available. Ultrasound offers excellent temporal and spatial resolution and the possibility to quantify flow. However, parts of the great vessels often remain hidden due to the lack of acoustic windows. Computed tomography (CT) is considered the gold standard in aortic assessment but increases the radiation-induced lifetime risk of cancer mortality [5]. High rates of repeated imaging are considered problematic, particularly in younger patients [6], given the potential cumulative side effects of CT scanning. Moreover, CT relies on iodine-based contrast agents, increasing risks in patients with impaired renal function [7].

Magnetic resonance imaging (MRI) may be the preferred approach for avoiding radiation exposure. MRI enables flow quantification but is not constrained by the lack of acoustic windows that limits ultrasound. Classic gadolinium-based contrast-enhanced magnetic resonance angiography (CE-MRA) delivers excellent spatial and contrast resolution with short scan times. The quality of this technique strongly depends on the proper timing of contrast injection and data acquisition and the patient’s breath-hold capabilities, limiting its use to compliant patients [8].

In addition, the repetitive use of contrast agents may lead to allergic reactions, brain retention, or nephrogenic systemic sclerosis in end-stage renal disease [9–13]. Finally, the achievable spatial resolution in CE-MRA is limited by bolus timing and breath-hold duration. In addition, several non-contrast magnetic resonance angiography (MRA) sequences have been developed, such as electrocardiogram (ECG)-gated fast spin-echo, balanced steady-state free precession (bSSFP), spoiled gradient echo, and black blood MRI [14, 15].

Fat suppression can further improve MRA readability and presentation. In bright blood imaging, missing or insufficient fat suppression may lead to inadequate discrimination between the vessel lumen and surrounding fat. While spectral fat saturation can be limited by its high sensitivity to magnetic field inhomogeneities, robust Dixon water-fat separation has been proven effective in cardiovascular imaging [16–19]. In addition, compressed sensing reduces image noise and enables reduced acquisition times due to a sparse representation of the acquired object, a pseudo-random sub-sampling of k-space, and a nonlinear iterative image reconstruction [20, 21]. Both techniques may aid in overcoming the known drawbacks of the widely used bSSFP sequences: sensitivity to off-resonance effects and steady-state disruptions [22].

This study’s primary objective was to assess a newly developed non-contrast, flow-independent, 3D isotropic MRA sequence that combines respiratory compensation, ECG-triggering, compressed sensing, and Dixon water-fat separation at 3 T for the chest and abdomen. This MRA sequence was compared to ECG-gated high-pitch CT angiography (CTA), which is currently considered the clinical gold standard for aortic imaging. This study focused on achieving consistent aortic diameter measurements as its primary endpoint, while its secondary endpoint involved assessing subjective image quality.

## Materials and methods

### Patients

The local Institutional Review Board approved the study protocol (approval number: 59_21B), which complies with the Health Insurance Portability and Accountability Act criteria and the Declaration of Helsinki. All patients signed an informed consent form before study enrolment.

We retrospectively screened patients who underwent aortic CT examinations with third-generation dual-source CT (Somatom Force; Siemens Healthcare GmbH, Forchheim, Germany) over 15 months (January 2020 to May 2021) for participation in this study, identifying 168 patients. The exclusion criteria were death (*n* = 3) or debilitating illness (*n* = 21), age < 18 years (*n* = 3), colonization with multi-drug resistant microbes (*n* = 4), residential distance > 100 km from the hospital (*n* = 6), metallic aortic foreign materials (*n* = 34), or contraindications for MRI (i.e., claustrophobia or unsafe implants; *n* = 12). We contacted the remaining 85 patients by telephone and letter, of which 25 agreed to participate in this study. We did not ask for the reasons for non-participation to respect the patient’s privacy.

The study population comprised eight women and 17 men (mean age: 62 ± 13 years). Initial indications for CT were suspected aortic dissection (*n* = 14), aortic aneurysm (*n* = 5), and workup before planned interventions, such as transcatheter aortic valve implantation or percutaneous coronary intervention (*n* = 3), unknown structure at the aortic root (n = 1), known aortic dissection (*n* = 1), and aortic coarctation (*n* = 1).

At study entry, the participants’ mean weight was 90 ± 22 kg, mean height was 174 ± 11 cm, and mean body mass index was 30 ± 6 kg/m^2^. Their mean heart rate during the MRI exam was 68 ± 8 beats/min.

### MRI protocol

Cardiovascular MRI was performed on a 3-T MRI system (MAGNETOM Vida; Siemens Healthcare, Erlangen, Germany) with dedicated phased-array receiver coils (an 18-channel body coil array and 32-channel spine coil array). A single coil array was used for the thoracic aorta, and when clinically indicated, a second coil array was added to assess the abdominal aorta.

The prototypical native MRA acquisition was planned as a coronal slab. The number of slices was adapted individually to include the entire aorta and heart. Geometrical parameters included a field of view of 450 × 450 × 156 ± 24 mm^3^ and an acquired and reconstructed resolution of 1.2 × 1.2 × 1.2 mm^3^. A two-point Dixon with echo times of 1.3 or 2.9 ms was used. An undersampling factor of *R* = 11 was achieved using a Poisson-disc-like incoherent sampling pattern combined with an iterative compressed sensing reconstruction approach. An adiabatic T2-preparation pulse was used for optimal vessel contrast. Prospective ECG-gating in an end-diastolic phase was used to avoid heart motion. A cross-beam navigator was placed onto the liver dome, and the position of the liver-diaphragm interface was tracked before every acquisition window to mitigate artifacts from respiratory motion. An accept-reject algorithm was applied with an acceptance window of ± 4 mm at end-expiration. All images were reconstructed inline in the MRI system.

### CTA protocol

CTA imaging was performed on a third-generation dual-source scanner (SOMATOM Force; Siemens Healthcare, Erlangen, Germany) covering the entire thorax and abdomen. The scans were prospectively ECG triggered in the high-pitch mode (collimation = 2 × 192 × 0.6 mm, pitch = 3.2), and data were only acquired during end-diastole to avoid heart motion. For each patient, an arterial phase acquisition with bolus triggering in the ascending aorta was performed in the head-feet direction. When clinically indicated, an additional venous phase acquisition in the head-feet direction was performed 20 s after the first pass. Automatic tube current modulation (Care Dose 4D; reference = 180–204 mAs, depending on patient girth) and automatic tube voltage selection (Care kV; reference = 120 kV) were used for all patients. A total of 50–80 mL (mean = 64.7 mL ± 11 mL) of iodinated contrast agent (Imeron 350, Bracco, Milan, Italy), followed by 50 mL of 0.9% NaCl, were administered through a peripheral indwelling cannula at the right or left upper extremity at a 4 mL/s flow rate. Images were reconstructed in 0.6 mm slices in the transversal plane using smooth (Bv40) kernels using iterative reconstructions.

### Measurements

A dedicated vascular image interpretation workflow supported the evaluation of the aortic diameters (CT Vascular; Syngo.via VB50; Siemens Healthcare GmbH, Erlangen, Germany) with automated centerline definitions. Manual corrections of the centerlines were added by the readers when necessary. The effective inner diameter and cross-sectional area were documented at predefined aortic levels (i.e., aortic annulus, sinus of Valsalva, sinotubular junction, mid ascending aorta, proximal aortic arch, mid aortic arch, proximal descending aorta, mid descending aorta, diaphragm level, celiac trunk level, and above the bifurcation) [2].

### Image quality analysis

Two board-certified reviewers ([blinded for review], with 10 and 7 years of experience in cardiovascular imaging, respectively), who were blinded to all clinical and imaging data, evaluated the datasets. The presentation started in the original orientation, and images were reviewed in free multiplanar reformation angulations at the discretion of the raters using a dedicated three-dimensional (3D) viewer (Horos v. 3.3.6; distributed under the LGPL license by Horosproject.org).

Overall image quality and water-fat separation were rated on a five-point scale [23]: 5 = excellent image quality, interpretable with no artifacts; 4 = good image quality, interpretable with minimal artifacts; 3 = average image quality, interpretation mildly degraded by image artifacts; 2 = below average image quality, interpretable but moderately degraded; 1 = poor image quality, uninterpretable images. The image quality of the myocardium, the aorta at the 11 abovementioned predefined levels, the thoracic and abdominal side branches, the pulmonary arteries, and the vena cavae were evaluated on a similar scale (Table 1). In cases of impaired image quality, the reason was documented in each case.

**Table 1:** Summary of subjective image quality scores. Values are given as median (range) and interquartile range (IQR)

	MRA	CTA	P
Overall image impression	5 (range 4–5); IQR 5–5	5 (range 4–5); IQR 5–5	0.71
Myocardium	5 (range range 3–5); IQR 4–5	5 (range 3–5); IQR 3–5	0.42
Aortic annulus	5 (range 4–5); IQR 4.75–5	5 (range 3–5); IQR 5–5	0.74
Sinus of Valsalva	5 (range 4–5); IQR 5–5	5 (range 3–5); IQR 5–5	0.91
Sinotubular junction	5 (range 4–5); IQR 5–5	5 (range 3–5); IQR 5–5	0.71
Mid ascending aorta	5 (range 4–5); IQR 5–5	5 (range 3–5); IQR 5–5	0.71
Proximal aortic arch	5 (range 3–5); IQR 5–5	5 (range 4–5); IQR 5–5	0.26
Mid aortic arch	5 (range 4–5); IQR 5–5	5 (range 3–5); IQR 5–5	0.63
Proximal descending aorta	5 (range 5–5); IQR 5–5	5 (range 4–5); IQR 5–5	0.32
Mid descending aorta	5 (range 5–5); IQR 5–5	5 (range 4–5); IQR 5–5	0.38
Aorta at hiatus level	5 (range 4–5); IQR 5–5	5 (range 4–5); IQR 5–5	0.32
Aorta at celiac level	5 (range 4–5); IQR 5–5	5 (range 4–5); IQR 5–5	0.19
Above aortic bifurcation	5 (range 4–5); IQR 5–5	5 (range 5–5); IQR 5–5	Sample size too small
Left coronary artery	5 (range 3–5); IQR 4–5	5 (range 3–5); IQR 5–5	0.39
Right coronary artery	5 (range 0–5); IQR 3–5	5 (range 1–5); IQR 4–5	0.47
Supra–aortic vessels	5 (range 2–5); IQR 4–5	5 (range 3–5); IQR 5–5	0.07
Celiac trunc	5 (range 3–5); IQR 5–5	5 (range 4–5); IQR 5–5	0.25
AMS	5 (range 3–5); IQR 5–5	5 (range 4–5); IQR 5–5	0.48
Left renal artery	5 (range 3–5); IQR 4.5–5	5 (range 2–5); IQR 5–5	0.48
Right renal artery	5 (range 3–5); IQR 5–5	5 (range 4–5); IQR 5–5	0.71
Pulmonary arteries	5 (range 3–5); IQR 4–5	5 (range 1–5); IQR 2–5	0.05
Pulmonary veins	1 (range 1–5); IQR 1–2	5 (range 1–5); IQR 5–5	< 0.0001
IVC *n*_*CT*_ = *17*	3 (range 1–5); IQR 3–4 n_25_: 4 (range 1–5); IQR 3–4	2 (range 1–5); IQR 1–3	< 0.0001
SVC *n*_*CT*_ = *17*	5 (range 4–5); IQR 4–5	3 (range 3–5); IQR 3.75–5	0.0003

### Statistical analysis

Interval-level data were evaluated for normality using the Shapiro–Wilk test. Data are presented as mean ± standard deviation (SD) or median (range). Data were compared using a paired *t*-test or Wilcoxon’s signed-rank test. A *p*-value < 0.05 was considered statistically significant. Bland–Altman plots and box plots were analyzed. Inter-rater agreement was evaluated using Cohen’s kappa value (κ), with κ interpreted as follows [24]: 0 < κ ≤ 0.2 = slight agreement, 0.2 < κ ≤ 0.4 = fair agreement, 0.4 < κ ≤ 0.6 = moderate agreement, 0.6 < κ ≤ 0.8 = substantial agreement, 0.8 < κ < 1.0 = almost perfect agreement, and κ = 1.0 as perfect agreement. All statistical analysis was performed using MedCalc Statistical software (version 20.218; MedCalc Software Ltd, Ostend, Belgium).

## Results

The interval between the initial CTA and additional MRA scans was 7 ± 5 months. The MRA acceptance rate was 42.7% ± 12.4%, leading to total net acquisition times of 08:04 ± 02:52 min. Four patients presented with cardiac arrhythmia during the MRA scan but were not excluded from this study. The observed arrhythmias types were bigeminal ventricular extrasystoles, frequent undefined ventricular extrasystoles, atrial fibrillation, and severe sinus arrhythmia. One patient presented with low voltage and a flattened ECG due to obesity, with repeated trigger failure observed during the scan (190 cm, 150 kg).

The mean CT scan time was 0.90 ± 0.19 s. The median tube voltage was 100 kV (100–120 kV), and the mean tube current was 352.6 ± 88.7 mAs. For arterial phase CT only, the CT dose index volume reached 4.6 ± 2.0 mGy, and the dose length product (DLP) reached 299.3 ± 140 mGy*cm. The DLP almost doubled to 519.1 ± 230.0 mGy*cm for the combined arterial and venous phase.

The final diagnoses are shown in Table 2.

**Table 2:** Summary of the evaluated aortic diseases

Normal/exclusion of aortic dissection	15
Aortic aneurysm	7
Aortic coarctation repair	1
Stanford B dissection	1
Lusoria artery	1

### Aortic measurements

**Fig. 1:**
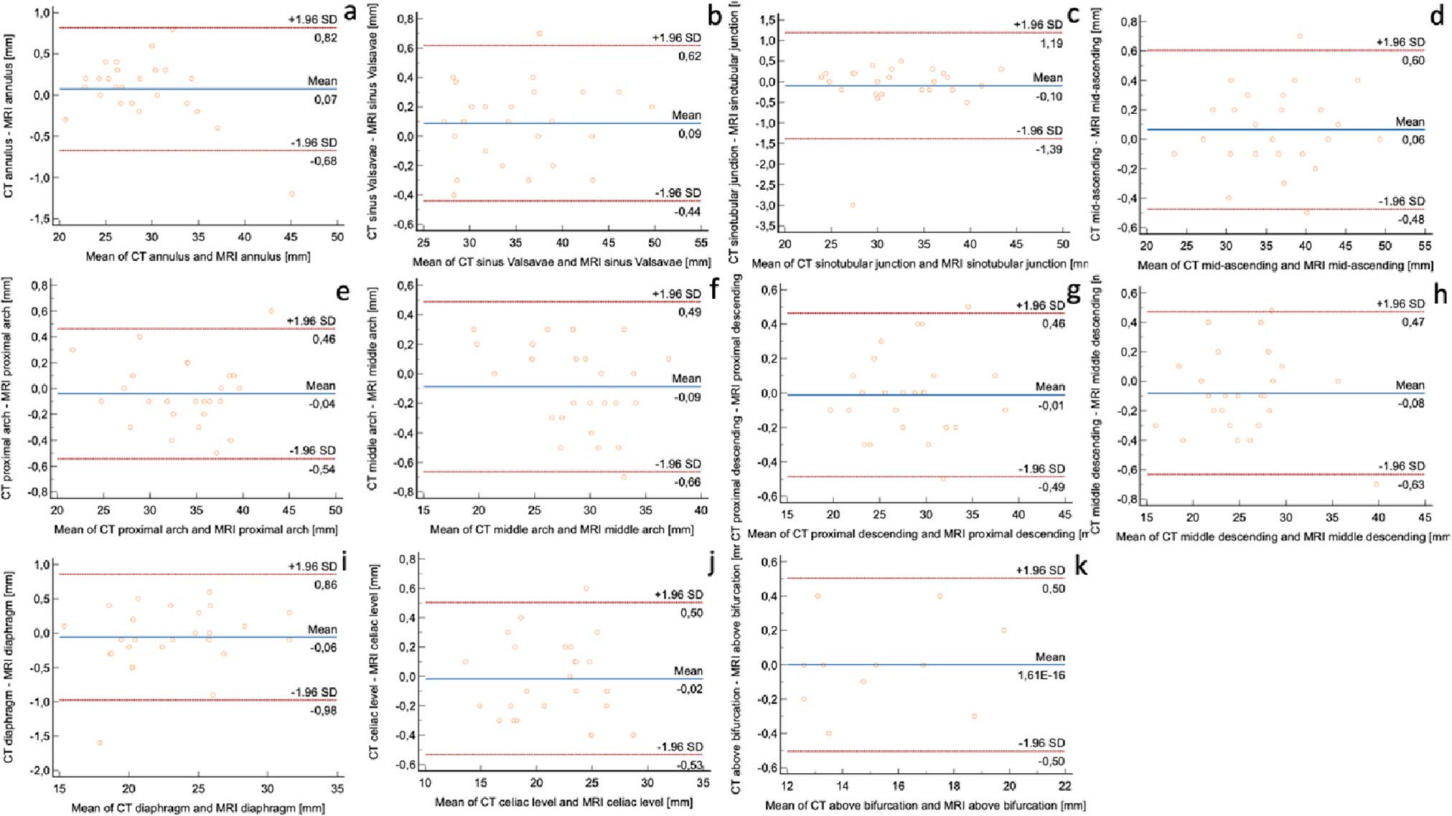
Bland–Altman-plots of the 11 standardized aortic segments: (**a**) aortic annulus, (**b**) sinus of Valsalva, (**c**) sinotubular junction, (**d**) mid ascending aorta, (**e**) proximal aortic arch, (**f**) mid aortic arch, (**g**) proximal descending aorta, (**h**) mid descending aorta, (**i**) diaphragm level, (**j**) celiac trunk level, and (**k**) above the bifurcation. The difference between the effective diameters of CTA and MRA is shown on the y-axis. The mean of the effective diameters is given on the x-axis. The limits of agreement (± 1.96* SD), the equality line, and the mean difference are indicated. The differences were generally < 0.5 mm, consistent with other studies [25, 26]

No significant difference was found between native MRA and contrast-enhanced CTA for all aortic segments (*p* = 0.08–0.94). Detailed measurement results are shown in Fig. 1 and Table 3.

**Table 3:** Detailed results comparing computed tomography (CT) and magnetic resonance (MR) datasets at predefined aortic levels

	Mean	SD	95% confidence interval	*p*	Mean difference	SD mean difference
MRA annulus D_eff_	28.8	5.3	2.1	0.12	−0.1	0.4
CTA annulus D_eff_	28.9	5.2	2.0			
MRA annulus area	674.9	276.9	108.5	0.10	−4.9	17.2
CTA annulus area	679.8	275.7	108.1			
MRA sinus of Valsava D_eff_	35.2	6.1	2.4	0.12	−0.1	0.3
CTA sinus of Valsava D_eff_	35.3	6.1	2.4			
MRA sinus Valsavae area	1013.5	362.9	142.3	0.56	2.7	4.6
CTA sinus Valsavae area	1010.8	364.4	142.9			
MRA sinotubular junction D_eff_	32.2	5.3	2.1	0.45	0.1	0.7
CTA sinotubular junction D_eff_	32.1	5.4	2.1			
MRA sinotubular junction area	835.7	274.3	107.5	0.78	−0.8	13.9
CTA sinotubular junction area	836.5	275.7	108.1			
MRA mid-ascending D_eff_	36.3	6.1	2.4	0.26	−0.1	0.3
CTA mid-ascending D_eff_	36.4	6.2	2.4			
MRA mid-ascending area	1062.5	348.4	136.6	0.89	−0.5	16.8
CTA mid-ascending area	1063.0	352.2	138.1			
MRA proximal arch D_eff_	33.7	5.1	2.0	0.44	0.04	0.3
CTA proximal arch D_eff_	33.7	5.1	2.0			
MRA proximal arch area	902.9	262.4	102.9	0.33	3.9	18.1
CTA proximal arch area	899.3	268.1	105.1			
MRA mid arch D_eff_	28.9	4.4	1.7	0.15	0.1	0.3
CTA mid arch D_eff_	28.8	4.3	1.7			
MRA mid arch area	672.6	191.8	75.2	0.08	5.2	14.2
CTA mid arch area	667.3	188.1	73.7			
MRA proximal descending D_eff_	28.3	4.7	1.9	0.81	0.01	0.2
CTA proximal descending D_eff_	28.3	4.7	1.9			
MRA proximal descending area	672.8	304.5	119.4	0.70	0.8	11.4
CTA proximal descending area	672.0	305.1	119.6			
MRA mid descending D_eff_	25.5	5.1	2.0	0.16	0.1	0.3
CTA mid descending D_eff_	25.4	5.0	2.0			
MRA mid descending area	532.7	230.3	90.3	0.12	2.5	10.2
CTA mid descending area	530.3	229.8	90.1			
MRA hiatus D_eff_	23.1	4.1	1.6	0.53	0.1	0.5
CTA hiatus D_eff_	23.0	4.2	1.6			
MRA hiatus area	430.8	154.1	60.4	0.38	4.3	23.9
CTA hiatus area	426.5	159.7	62.6			
MRA celiac level D_eff_	21.4	3.9	1.5	0.76	0.02	0.3
CTA celiac level D_eff_	21.4	3.9	1.5			
MRA celiac level area	370.0	128.7	50.4	0.29	3.1	13.9
CTA celiac level area	367.0	129.3	50.7			
MRA above bifurcation D_eff_	15.3	2.4	1.0	0.30	−0.5	6.4
CTA above bifurcation D_eff_	15.3	2.5	1.0			
MRA above bifurcation area	187.5	61.1	25.0	0.94	−0.002	0.2
CTA above bifurcation area	187.9	62.3	24.4			

*D*_*Deff*_ effective diameter [mm], *area*   cross-sectional area [mm^2^]

### Image quality

The visual assessments of general image quality, myocardium, the various aortic segments, and proximal branches showed no significant differences between the two methods (*p* = 0.07–0.91). Similarly, there was no significant distinction in the evaluation of pulmonary arteries between the two methods (*p* = 0.05). Both raters favored MRA for the superior and inferior vena cava (IVC; both *p* < 0.05). The rating of the pulmonary veins was better in the CTA acquisitions (*p* < 0.05). Inter-rater agreement was at least substantial in all cases (κ > 0.7).

Overall, MRA image quality was downgraded by one point in 4/25 (16%) cases due to uncorrected vessel motion (2/25, 8%) or respiratory motion artifacts (2/25, 8%). We found flow voids in 12/437 (3%) of the systemic arterial segments (aortic segments and branches), 3/25 (12%) of the pulmonary artery segments, and 47/50 (94%) of the systemic venous segments. In 4/25 cases, the MRA images had a “systemic arterial” weighting with low contrast in the pulmonary arteries and systemic veins.

In the four patients with complex ECG findings, the image quality was downgraded in the myocardium (grade 3 in 1/4 cases, grade 4 in 2/4 cases), aortic annulus (grade 4 in 2/4 cases), sinus of Valsalva (grade 4 in 1/4 cases), right and/or left coronary artery (grade 2 in 1/8 cases, grade 3 in 3/8 cases), sinotubular junction and ascending aorta (both grade 4 in 1/4 cases), and pulmonary arteries (grade 2 in 1/4 cases). Otherwise, the image quality was generally excellent.

An excellent fat suppression (score 5) was achieved in 24/25 (96%) cases. One patient had a fat–water swap separating the two body halves.

Overall, CTA image quality was reduced by one point in 4/25 (16%) cases as a result of reduced aortic contrast due to bolus timing (3/25, 12%) and/or off-center artifacts due to patient positioning (2/25, 8%). The infrarenal inferior vena cava was rated with low diagnostic confidence, or worse, in the venous phase images in 12/17 cases (71%) due to insufficient vessel contrast.

**Fig. 2:**
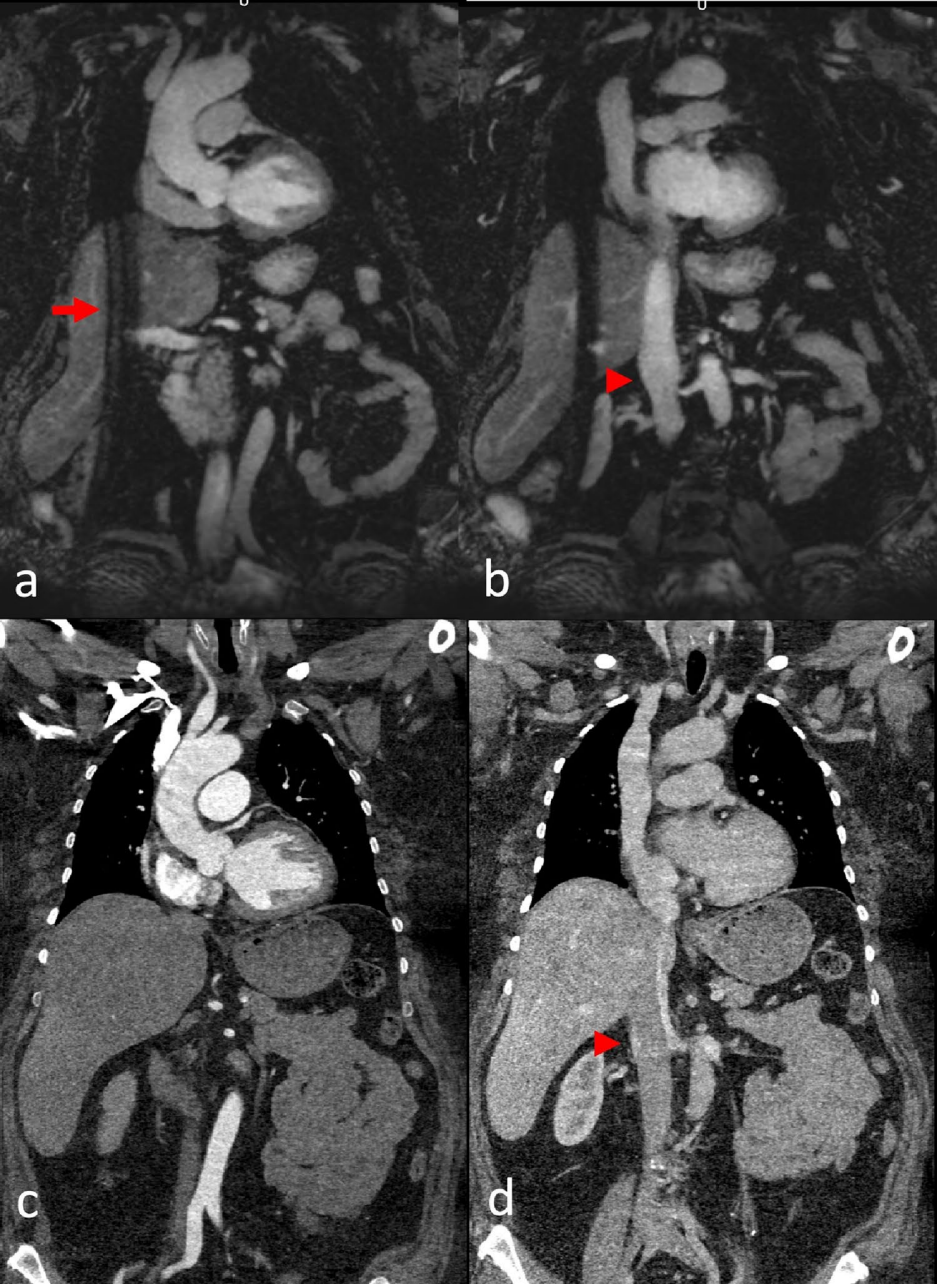
This patient was admitted to the Emergency Department for acute thoracic pain and a difference in pulse between arms. The initial CT scan ruled out aortic dissection. Native coronal MRA images (**a**, **b**) and corresponding CTA reformations (**c**, **d**) at the level of the ascending aorta (**a**, **c**) and venae cavae (**b**, **d**). A typical MRA navigator artifact is seen over the right hemibody (arrow). There was insufficient contrast in the IVC in the CTA due to an influx of contrasted blood from the renal veins but excellent contrast in the MRA (arrowheads)

**Fig. 3:**
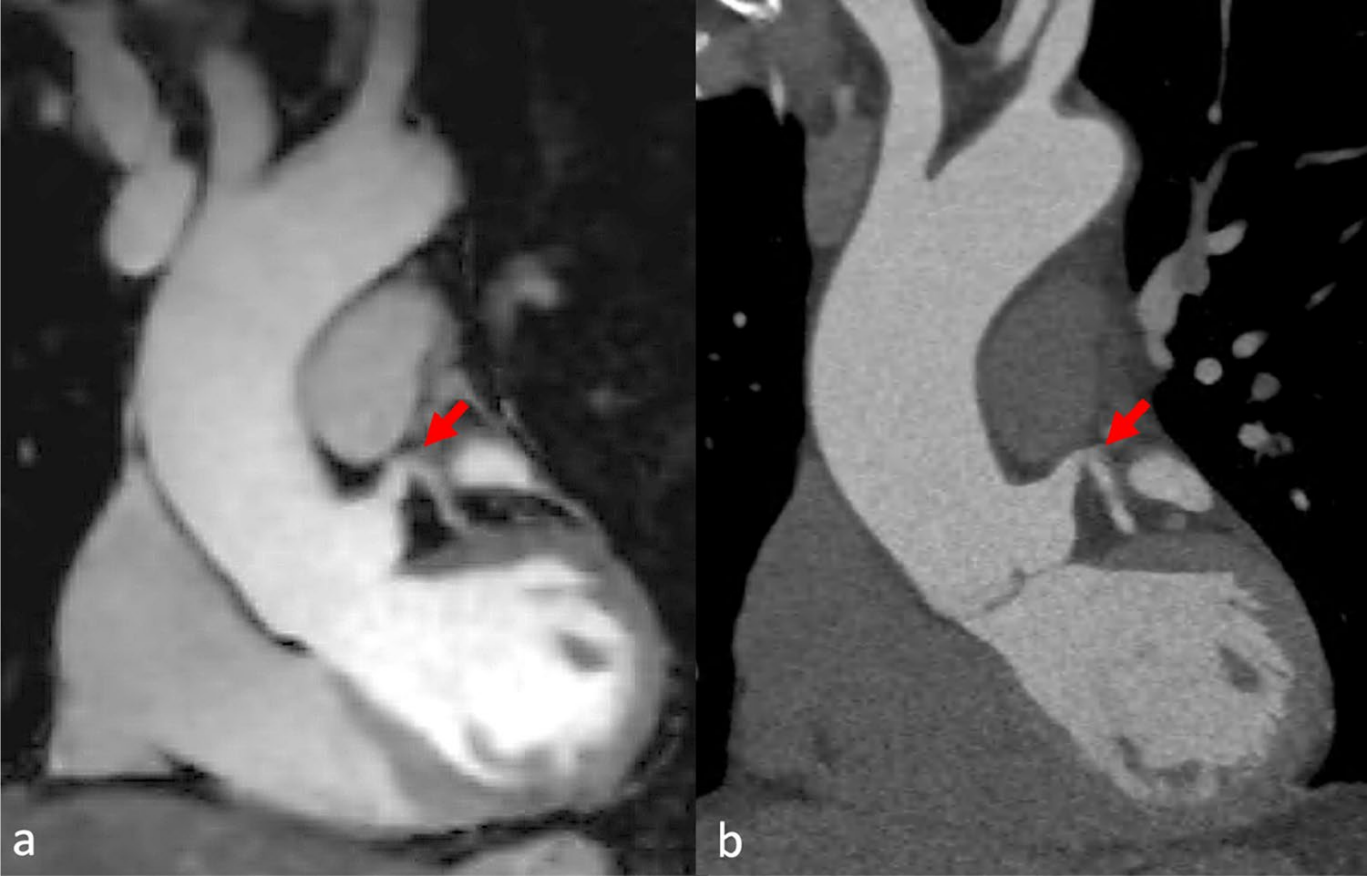
A patient had an unclear structure at the aortic root noted in the echocardiography. The initial CT scan was performed to confirm the findings, but it did not reveal any abnormalities. Coronal reformation of MRA (**a**) and CTA (**b**) of the aortic root and ascending aorta. Excellent depiction of the proximal left coronary artery (arrow)

**Fig. 4:**
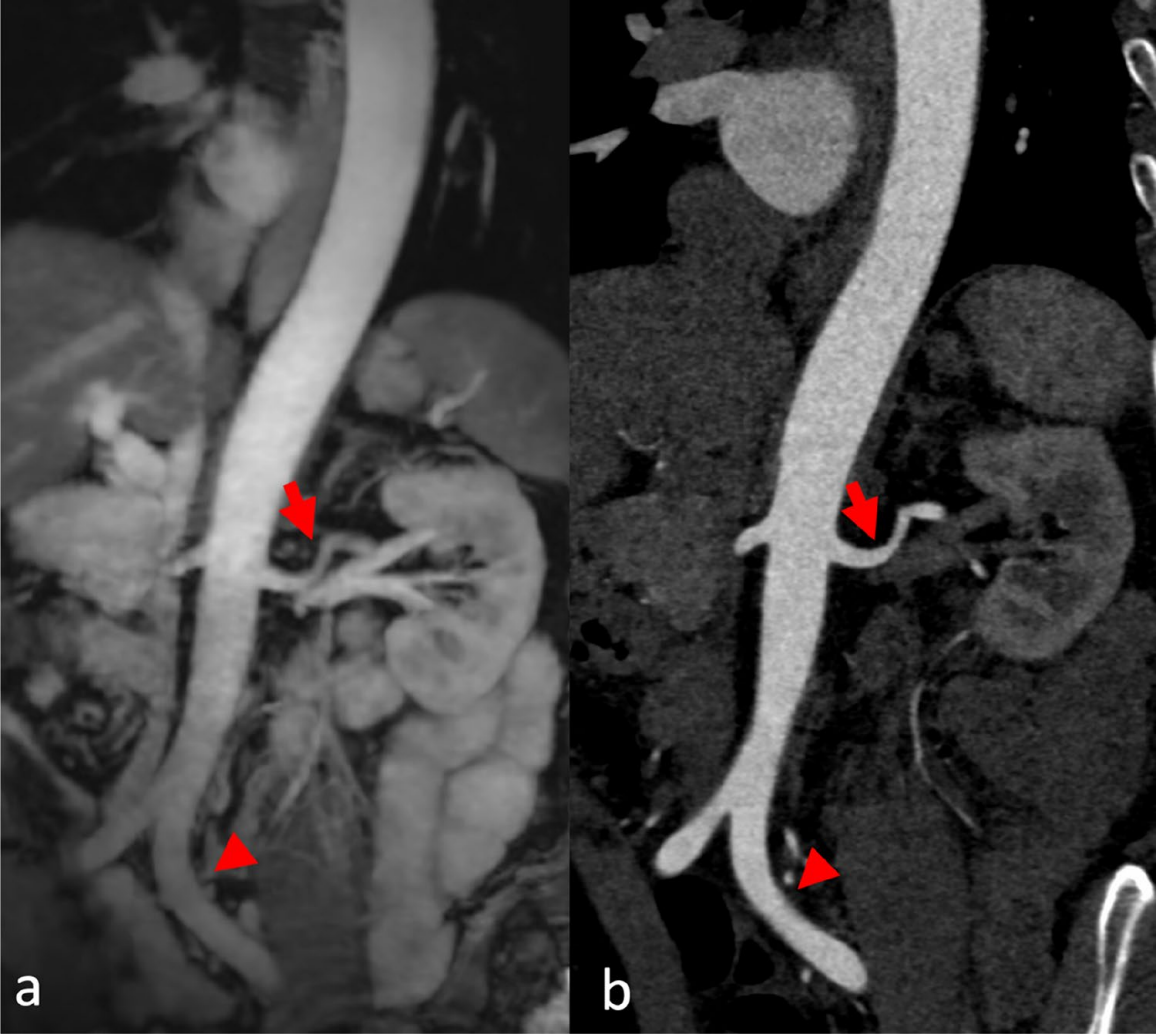
A female patient had experienced multiple collapses on the day of admission and presented to the Emergency Department with cold, clammy skin and severe thoracic pain. The aortic CT scan ruled out dissection. Coronal reformation of MRA (**a**) and CTA (**b**) of the abdominal aorta to the iliac arteries (arrowhead). Excellent depiction of smaller vessels, such as the left renal artery (arrow)

**Fig. 5:**
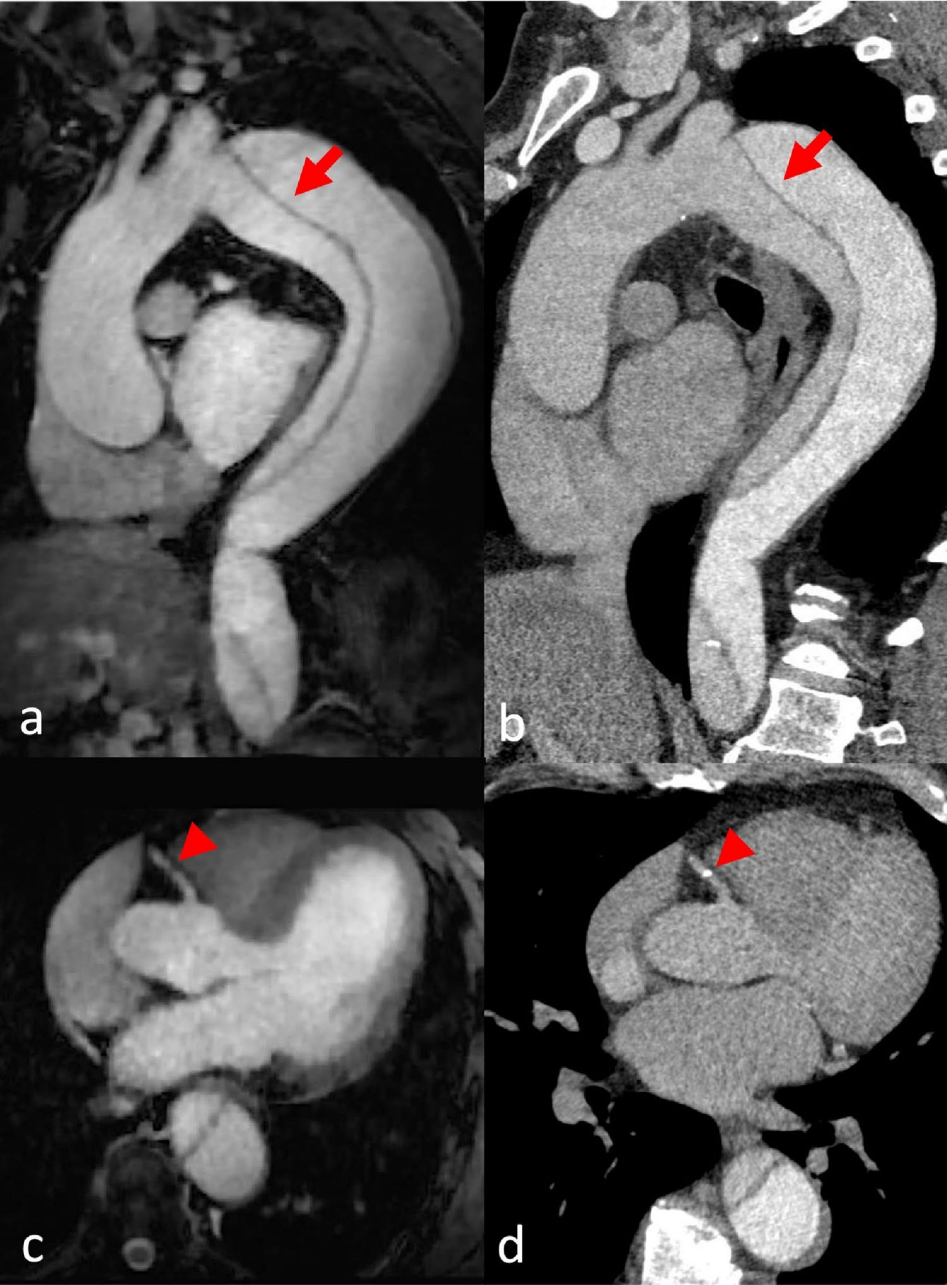
A patient with Stanford B aortic dissection was scheduled for follow-up imaging. Sagittal (**a**, **b**) and axial (**c**, **d**) reformations of MRA (**a**, **c**) and CTA (**b**, **d**). Good depiction in both modalities with unusual bolus timing in the CTA (**a**) due to a misplaced region of interest in the false lumen (arrow). Flow insensitivity renders both lumina hyperintense in MRA (**b**). A small calcification was found in the right coronary artery (arrowhead)

**Fig. 6:**
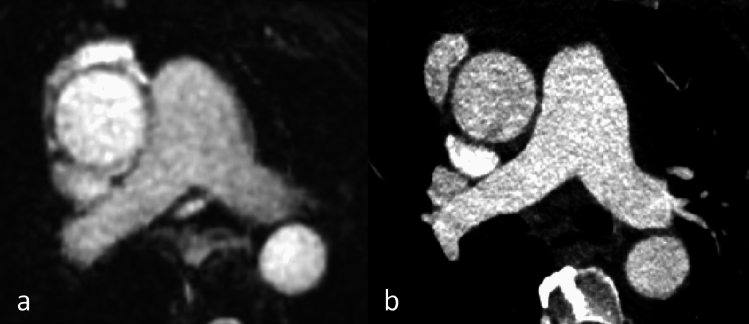
A patient presented after a near-collapse episode at rest. Hypotension and a blood pressure difference between arms were observed in the Emergency Department. The CT scan ruled out aortic dissection. Axial reformation of an enlarged view of the pulmonary arteries in MRA (**a**) and CTA (**b**). Excellent image quality in both modalities despite a lower resolution in the MRA (**a**)

Example MRA and CTA images are provided in Figs. 2, 3, 4, 5 and 6, and detailed image quality scores are provided in Table 1.

## Discussion

This study evaluated a novel, large field-of-view, highly accelerated, navigator- and ECG-gated 3D native MRA sequence with Dixon water-fat separation. We compared it with a state-of-the-art ECG-triggered aortic CTA provided by a third-generation dual-source scanner.

Despite the relatively long interval of 7 ± 5 months, the diameter and cross-sectional area in the various aortic segments did not differ significantly between the two methods. Most disagreements were < 0.5 mm, consistent with other comparisons of aortic CTA and MRA [25, 26]. Three outliers between 1.5 and 3.0 mm at the level of the sinotubular junction and the diaphragm may have occurred due to discrepancies in the measurement level or true dilatation between the different time points. Nevertheless, these differences are not clinically significant because the threshold growth in current guidelines is 5 mm [2].

Both methods generally delivered excellent image quality. The image quality of the aorta, its branches, and the pulmonary arteries was rated similarly in both modalities. MRA outperformed venous phase CTA for the superior and inferior venae cavae due to lower vascular contrast in the CTA images, particularly in the infrarenal vena cava. Interobserver agreement was at least substantial (*κ* > 0.7).

With acquisition times of 08:04 ± 02:52 min vs. 0.90 ± 0.19 s, the MRA acquisition was significantly longer than the CTA acquisition. Nevertheless, this acquisition time was comparable to other thoracic aortic MRA studies, which reported acquisition times of 05:56 min to 08:06 min for thoracic aortic MRA alone at a lower resolution of 1.3–1.5 mm^3^ [18, 27, 28]. The proposed sequence covers a large field of view that includes nearly the entire chest and abdomen, down to the aortic bifurcation, at a higher resolution of 1.2 mm^3^. This coverage is comparable to CTA as a reference technique and superior to the published results on the thoracic aorta in a parasagittal orientation [29]. The Dixon technique proved to be largely insensitive to magnetic field inhomogeneities, with robust water-fat separation in all but one case. The iterative compressed sensing reconstruction, performed inline on the scanner, took approximately 1 min. All MRA datasets were diagnostic despite the relatively challenging conditions, including patients suffering from arrhythmia and obesity (weight up to 150 kg), and the sequence proved robust for routine clinical scanning.

A similar sequence, modified relaxation-enhanced angiography without contrast and triggering (modified REACT), was previously compared with a non-ECG-gated contrast-enhanced MRA, showing superior image quality from the aortic annulus to the mid-ascending aorta [18]. Compared to 2D steady-state free precession imaging of the aorta, the sequence showed comparable vessel diameters while offering the possibility of 3D reformations [30]. We compared the proposed MRA sequence to state-of-the-art ECG-triggered aortic CTA provided by a third-generation dual-source scanner as the current clinical gold standard in non-invasive aortic imaging.

The proposed MRA sequence is unsuitable for emergencies due to the prolonged scan time and limited patient surveillance during acquisition. In most other cases, we consider it a valuable alternative that avoids exposure to ionizing radiation and contrast agents, especially in patients who undergo regular examinations. Many such patients are young and have congenital aortic defects or diseases such as Marfan syndrome and are, therefore, particularly prone to developing radiation-induced cancer [6]. Conversely, atherosclerotic aortic lesions often co-exist with multisystemic effects, including impaired renal function, which may be potentially exacerbated by iodine-based contrast agents [31]. Moreover, CTA demands high-contrast injection rates of 4–5 mL/s [32], a known risk factor for extravasation [33]. Due to native acquisition, there is no risk of allergic reactions due to contrast agents, which may be especially beneficial for patients with known severe adverse reactions to iodinated or gadolinium-based contrast agents. Patients unable to follow respiratory pauses may also benefit from the robust MRA technique in free breathing. Given its flow independence, all major thoracic and abdominal vessels can be evaluated in a single examination rather than a multiple-scan protocol with a multiple-dose application. This advantage may be particularly beneficial in assessing a complex post-surgery event such as Fontan circulation or atrial and arterial switch operations.

## Limitations

This study had some limitations that should be mentioned. First, it excluded patients with aortic implants; studies including this patient cohort should be performed to evaluate the performance of the proposed sequence in the presence of metallic implants. Second, the time interval between the initial clinically indicated CTA and the MRA was relatively long. Third, there was a potential selection bias for more compliant patients due to the relatively low positive response rate of 25 participants out of 83 contacted patients. Fourth, the sample size was relatively small (*n* = 25). Fifth, the contrast bolus volume in the CTA was optimized for aortic imaging; therefore, the image quality of the systemic veins may have been compromised in the venous phase images. Sixth, image quality was assessed primarily based on subjective scales. Finally, not every hospital has an MRI device, especially at 3 T, while CT devices are much more widespread.

## Conclusion

This study’s novel, advanced MRA sequence provides excellent image quality and reliable measurements in routine and challenging patients. It avoids adverse effects such as extravasation, renal insufficiency, and stochastic radiation damage in vulnerable patients with cardiovascular diseases or malformations. The free-breathing technique improves patient compliance. Therefore, it is a perfect tool for repeated follow-up examinations.

## Data Availability

The data supporting the findings of this study are available upon reasonable request from the corresponding author. However, please note that we cannot make the complete image datasets publicly available since they contain personal information that could compromise the study participants’ privacy and consent.
